# Changes in patient satisfaction related to their perceived health state during implementation of improved integrated care for older persons

**DOI:** 10.1371/journal.pone.0216028

**Published:** 2019-05-16

**Authors:** Antonius J. Poot, Daisy M. Wopereis, Wendy P. J. den Elzen, Jacobijn Gussekloo, Jeanet W. Blom

**Affiliations:** Department of Public Health and Primary Care, Leiden University Medical Center, Leiden, The Netherlands; University of Antwerp, BELGIUM

## Abstract

Patient satisfaction with the general practitioner (GP) is lower in older persons with a higher level of complexity of health problems. This study investigates whether, in these older persons, changes in satisfaction with their GP, on receiving improved integrated care, is related to their perceived health state.Using the Integrated Systematic Care for Older People (ISCOPE) trial (aimed at improving person- centered integrated care) this study compared changes in satisfaction with the GP in older persons (aged ≥75 years) with a high level of complex health problems on receiving integrated care, stratified for perceived health state at baseline. Satisfaction with the GP was registered on a 5-point Likert scale. Perceived health state was estimated with the Older Persons and Informal Caregivers Survey-Composite End Point (TOPICS-CEP) at baseline, stratified into 33% percentiles. Differences in satisfaction change between the intervention and usual care/control groups (overall and stratified for perceived health state) are presented by percentages of ‘very satisfied’ participants and improving or deteriorating 1 or more points on the Likert scale. At baseline, the intervention (n = 151) and control group (n = 603) were mainly female (75%) and living alone (62%); mean age was 83 years. Medical status, perceived health state and characteristics of participants were similar. Overall, at baseline 44.4% of respondents in the intervention group were ‘very satisfied’ compared with 37.1% at follow-up, (difference -7.3%). In the control group, ‘very satisfied’ at baseline was 32% and at follow up 29.2% (difference -2.8%). The p-value for this difference in change is 0.56. After stratification for TOPICS-CEP the results were the same. In older persons with a high level of complexity of health problems, implementation of person- centered integrated healthcare did not influence their satisfaction with the GP, also not among those with the highest or lowest perceived health state.

## Introduction

Integrated and patient-centered care can be defined as: the organization and management of health services so that people get the care they need, when they need it, in ways that are user-friendly, achieve the desired results, and provide value for money [1]. This type of care is considered necessary and advantageous for patients with complex care needs [2–5]. This applies particularly to older patients because of the higher level of complexity of their care needs, and their increasing absolute numbers and proportion in the general population [1]. Despite that the evidence concerning the (cost) effectiveness of integrated and person-centered care interventions remains unclear, there is strong consensus about the need for implementation amongst care providers and policymakers [6–8].

In its 2006 policy paper on Integrated Care, the World Health Organization observed that the various stakeholders have different expectations of integrated care. In particular, patients expect integrated care to be seamless, smooth and easy to navigate [1]. Patient satisfaction is a complicated concept which partly reflects the realisation of these expectations [9–11]. In addition, satisfaction is influenced by patient characteristics such as age and gender, and also reflects communicative provider skills more than care characteristics or quality [12–14].

Despite reservations concerning the meaning of patient satisfaction, it is argued that only the patient can determine whether his/her needs and expectations have been met [8, 15]. Therefore, no doubt exists about the relevance of perceptions and satisfaction of patients for the design and delivery of integrated care [7, 16–18].

Our earlier study showed that, in older persons, patient *dis*satisfaction with general practitioner (GP) care increased with the complexity of health problems independently of age, gender and morbidity [13]. This raised the question whether the decreased satisfaction level was related more to the experienced health state of the patients themselves, or to the failure of the integration and patient-centeredness of the provided care to meet the expectations of this category of patients.

This study aims to address this question by investigating changes in the satisfaction of older patients during implementation of integrated and person-centered care in relation to their perceived health state. For this, we compared changes in general satisfaction with GP care between two groups of patients aged ≥ 75 years with a high level of complexity of care needs at baseline and at 12-month follow-up. One group received improved integrated and patient-centered care and another group received usual care. The analyses were stratified according to the perceived health state of the patients.

## Methods

### Study design and participants

This study is embedded in the Integrated Systematic Care for Older People (ISCOPE) study. The Medical Ethical Committee of the Leiden University Medical Center approved the study. The study was registered in the Netherlands Trial Register (NTR1946).

The ISCOPE study is a cluster randomized trial in which all persons aged ≥75 years in 59 general practices received a structured postal questionnaire with 21 questions on four health domains (functional, somatic, mental, social) [19]. Inclusion of practices was determined by the willingness of the involved GPs to participate and accept randomization. All 560 GPs in the region of Leiden, the Netherlands, were approached of which 104 were willing to participate (19%). The majority of the participating GPs (77.9%) worked in an urban environment. The number of registered patients aged 75 years and older per GP ranged from 12 to 479 with a median of 131 (IQR 66 to 210) [20].

The 59 practices were randomized into 30 intervention and 29 control practices by a complete consent pre-randomization design [21, 22]. In each of the intervention practices 10 patients with health problems in 3 or 4 domains were randomly selected in order to make a care plan. The sample size calculation of the ISCOPE study was based on the change in ADL-score on the Groningen Activity Restriction Scale after one year. This power calculation has been published previously [19].

**Fig 1:**
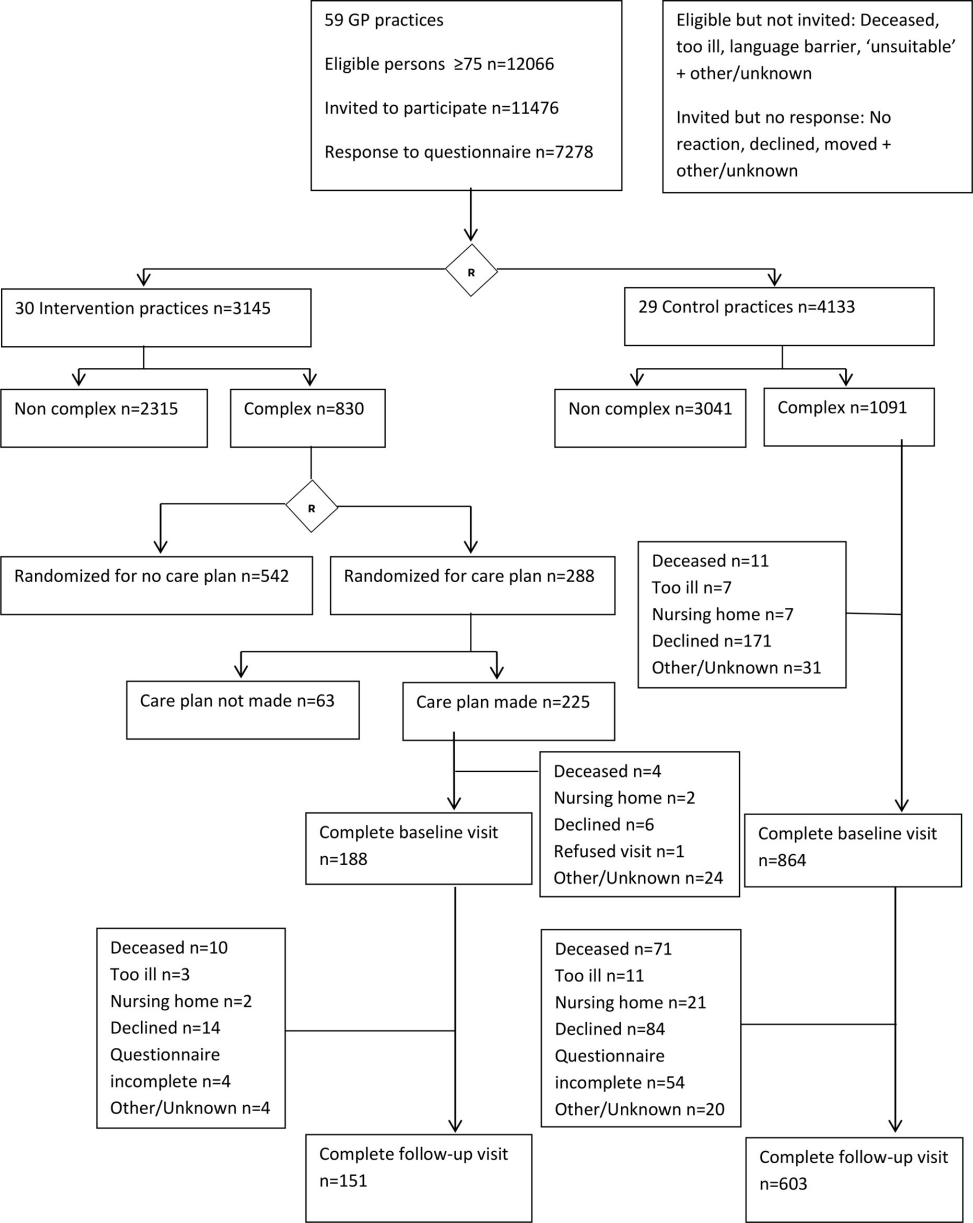
Flow chart showing the inclusion of study participants.

For the present study, the intervention group included all respondents to the postal questionnaire with health problems in 3 and 4 domains who: i) received a care plan, ii) answered the satisfaction questions, and iii) for whom a perceived health state score could be calculated. The usual care (control) group included respondents to the postal questionnaire with health problems in 3 and 4 domains who: i) received usual care in a control practice, ii) answered the satisfaction questions, and iii) for whom a perceived health state score could be calculated (Fig 1).

### Intervention

In the intervention practices, the GPs and practice nurses received training in making and performing a person-centered and integrated care plan for patients with complex problems. This 8 hour training included: i) accessing and using resources, and ii) organizing person-centered, proactive, multidisciplinary care for older persons in primary care. In the intervention practices the GP or practice nurse made a care plan for a maximum of 10 randomly chosen patients with problems in 3 or 4 domains as shown in Fig 1.

The care plan process was started by the GP or practice nurse making an inventory of the problems experienced by the older person in the somatic, activities of daily living (ADL), as well as in social, psychological and communicative areas. The wishes and expectations of the older person about goals to be achieved were explored in a dialogue with the participant and their informal caregiver(s). Actions, evaluation items and moments were formulated based on this dialogue. Other care professionals were involved when suggested by the care plan. During the intervention, the GPs had the possibility to consult another GP with special post-graduate training in geriatric care in general practice. Patients in the intervention practices who received a care plan were compared with patients with similar complexity (i.e. problems in 3 or 4 domains) who received usual care in the control practices.

### Outcomes and follow-up

At baseline and at 1-year follow-up, participants were visited by a research nurse to measure characteristics and outcomes. These included demographics, healthcare utilization, morbidities, functional limitations, emotional wellbeing, pain experience, cognitive problems, social functioning, self-perceived health, self-perceived quality of life (QOL), and satisfaction with their care providers.

Satisfaction with GP care was measured by asking: ‘*How satisfied or dissatisfied are you about your GP practice*?’ Responses were recorded on a 5-point Likert scale ranging from ‘very satisfied’, ‘satisfied’, ‘neutral’, ‘dissatisfied’ to ‘very dissatisfied’. We chose to express the aggregated satisfaction response as the percentage ‘very satisfied’ and the percentage rising/decreasing at least one category rather than a mean score, since the choice ‘very satisfied’ is the most meaningful and there is little effect size variation in the mean Likert score [23, 24].

Experienced health state was quantified at baseline using the TOPICS-MDS CEP. This measure was developed as a combined end point (CEP) of **T**he **O**lder **P**ersons and **I**nformal **C**aregivers **S**urvey **M**inimal **Data Set** (TOPICS-MDS) for the studies within the Dutch National Care for the Elderly Program, of which the ISCOPE study was part [25, 26]. The TOPICS-MDS CEP is an individual aggregation of the outcomes of all the used instruments indicating health and wellbeing, with a preference weight arrived at through a vignette study with a panel of older persons and informal caregivers[27]. For this, the instruments used are morbidity, functional limitations, emotional wellbeing, pain experience, cognitive functioning, self-perceived health and self-perceived QoL. It has been validated as a measure for evaluation of health state by older persons in various settings [28]. We used the TOPICS-CEP syntax to calculate the score for the individual participants in the ISCOPE intervention and control groups at baseline. The TOPICS-MDS CEP gives a score for perceived health state, ranging from 0 (worst possible perceived health state) to 10 (best possible perceived health state).

### Statistical analysis

To characterize and compare the intervention and control groups at baseline the following were calculated: i) median age, ii) number of diseases, and iii) percentage of participants who were female, living alone and had completed a higher education. To quantify activity restriction and cognitive impairment median scores were calculated using the Groningen Activities Restriction Scale (GARS) and Mini-mental State Examination (MMSE) respectively. The GARS has a minimum score of 18 and a maximum of 72, with higher scores indicating greater limitation and in the MMSE the maximum of 30 indicates no cognitive impairment and a score below 24 is considered indicative of dementia [29, 30]. Differences between the intervention and control groups at baseline were tested with a chi-square test for dichotomous variables and for continuous variables with a t-test for parametric scores and with the Mann-Witney U-test for non-parametric scores.

We defined two outcome measures as an expression of the change in satisfaction: i.e. we calculated between baseline and follow-up: 1) the change in percentage of participants who reported being ‘very satisfied’, and 2) the proportion of participants who showed an increase or decrease of 1 or more points on the Likert scale. The changes in satisfaction were compared between the intervention and usual care group, stratified for TOPICS-CEP; this was performed in three strata, each representing a third of the total group (i.e. low, middle and high TOPICS-CEP).

Analysis of the difference in change in percentage of very satisfied persons in the intervention and usual care groups at the follow-up measurement was adjusted for age, sex and clustering by practice using generalized estimating equation (for dichotomous outcomes). Values were calculated for the intervention and usual care groups, overall and per stratum of TOPICS-CEP. For the difference in the proportion of participants who showed an increase or decrease of 1 or more points on the likert scale, we used logistic regression models adjusted for age, sex and clustering per practice.

A p-value < 0.05 was considered statistically significant. All analyses were conducted with IBM SPSS Statistics for Windows version 20.0.

## Results

Sociodemographic, functional and medical characteristics, as well as perceived health state and satisfaction for the intervention and control group are presented in Table 1. Of all patients, 75% were female and 62% were living alone. The intervention group was younger than the control group (82.1 vs 83.2; p = 0.04). Slightly more participants in the intervention group had completed a higher education (70.2% vs 61.0% p = 0.05). The groups showed no differences in gender, living situation, number of diseases/ailments, activity restriction and cognitive impairment. The perceived health state at baseline quantified by TOPICS-CEP showed no significant difference between the intervention and control group (both scoring between 6 and 7). Differences in the distribution over the five satisfaction categories were not significant (p = 0.08).

**Table 1:** Baseline sociodemographic, medical and functional characteristics, perceived health state and satisfaction of the participants for the intervention and control group.

	Intervention group	Control group	P
	n = 151	n = 603	
Age: median (IQR)	82.1 (78.5–85.8)	83.2 (79.5–87.2)	0.04^¶^
Gender (n, (%))			0.84^#^
Female	112 (74.2)	452 (75.0)	
Male	39 (25.8)	151 (25.0)	
Living alone (n, (%))			0.94^#^
Yes	94 (62.3)	380 (63)	
No	57 (37.7)	223 (37.0)	
Level of education* (n, (%))			0.05^#^
Higher education	106 (70.2)	368 (61.0)	
Low education	45 (29.8)	235 (39.0)	
Number of diseases/ailments; median (IQR)	4 (3–5)	4 (3–6)	0.37^¶^
GARS score: median (IQR)	33 (27–43.3)	35 (28–43.3)	0.21^¶^
MMSE score: median (IQR)	28 (26–29)	28 (26–29)	0.89^¶^
TOPICS-CEP score: median (IQR)	6.9 (6.2–7.5)	6.8 (6.0–7.5)	0.13^¶^
Satisfaction with GP practice (n, (%))			0.08^#^
Very satisfied	67 (44.4)	193 (32.0)	
Satisfied	66 (43.7)	311 (51.6)	
Neutral	13 (8.6)	69 (11.4)	
Dissatisfied	4 (2.6)	25 (4.1)	
Very dissatisfied	1 (0.7)	5 (0.8)	

**GARS** the Groningen Activities Restriction Scale is a measure of activity restriction; range 18 to 72, higher scores indicating greater limitation; **MMSE** the Mini-mental State Examination is a measure of cognitive impairment; range 0 to 30, higher scores indicate less cognitive impairment; **TOPICS-CEP** The Older Persons and Informal Caregivers Survey Composite End Point is a measure of perceived health state; range from 0 to 10, higher scores indicate better perceived health state

IQR = inter quartile range

*Higher education = Completed continued education after secondary school

^¶^Mann-Witney U-test

^#^chi square test

Changes of satisfaction (expressed in differences of % ‘very satisfied’) between baseline and follow-up for the intervention and control group are shown in Table 2. Overall, at baseline 44.4% of respondents in the intervention group were ‘very satisfied’ compared with 37.1% at follow-up, resulting in a difference of -7.3%. In the control group, the difference between baseline and follow up was -2.8%. The p-value for this difference in change between the intervention and control groups calculated using the ‘Generalized estimation equation’ (GEE) method is 0.56. Within the strata of low, middle and high of TOPICS-CEP, these values are respectively -7.9 and -0.7 (p = 0.38), -11.4 and -1.4 (p = 0.68) and -1.9 and– 5.4 (p = 0.17).

**Table 2:** Changes in percentage of respondents that are very satisfied about GP care over 1-year follow-up during implementation of integrated care in the intervention group compared to control group, depending on perceived health state.

	Intervention group			Control group					
	n = 151			n = 603			Adjusted change in % change*		
	Baseline	Follow-up	% Change(95% CI) ^#^	Baseline	Follow-up	% Change(95% CI) ^#^	p	Β (SE)	Goodness of fit
Overall (n(%))	67 (44.4)	56 (37.1)	-7.3 (3.7;18.3)	193 (32.0)	176 (29.2)	-2.8(-10.8;2.4)	0.56	0.028(0.047)	351.252
TOPICS CEP strata									
Low 33%	16 (42.1)	13 (34.2)	-7.9(-26.9;11.1)	49 (25.8)	46 (24.2)	-0.7(-18.8;17.4)	0.38	0.071(0.077)	109.324
Middle 33%	29 (47.5)	22 (36.1)	-11.4(-30.6;7.8)	74 (34.9)	71 (33.5)	-1.4(-20.0;17.2)	0.68	-0.032(0.078)	123.452
High 33%	22 (42.3)	21 (40.4)	-1.9(-21.2;17.4)	70(34.8)	59(29.4)	-5.4(-23.7;12.9)	0.17	0.102(0.075)	122.538

* GEE, adjusted for baseline age, gender and cluster

^#^ CI = Confidence Interval

Similarly, the changes in satisfaction from baseline to follow-up between the intervention and control group are shown in Table 3; expressed as the percentage of respondents with a 1 or more point improvement or deterioration on the Likert scale. Overall, 26.5% of the intervention group improved 1 category or more in satisfaction vs. 25.4% in the control group (p = 0.68); a deterioration of 1 category or more occurred in 17.2% vs. 20.1% (p = 0.52), respectively. Similarly, in the low TOPICS CEP stratum, satisfaction improved in 26.3% in the intervention group vs. in 28.9% in the control group (p = 0.29), and deterioration in 21.0% vs. in 21.6%, respectively (p = 0.15). In the middle stratum, improvement was in 27.9% vs. 23.1% (p = 0.39), and deterioration in 13.1% vs. 20.8% (p = 0.75), respectively; and in the high stratum, improvement was in 25.0% vs. in 24.4% (p = 0.24), and deterioration in 19.2% vs. in 17.9% (p = 0.94), respectively.

**Table 3:** Changes in satisfaction about GP care over 1-year follow-up during implementation of integrated care in the intervention group compared to usual care (control), depending on perceived health state.

	Changes in satisfaction about GP care over 1 year follow-up^#^								
	Improve ≥ 1 category			No change			Deterioration ≤ 1 category		
	Intervention	Control	p*	Intervention	Control	p*	Intervention	Control	p*
Overall	26.5%(40 / 151)	25.4%(153 / 603)	0.68	56.3% (85 / 151)	54.6% (329 / 603)	0.37	17.2% (26 / 151)	20.1% (121 / 603)	0.52
TOPICS CEP strata									
Low 33%	26.3%(10 / 38)	28.9%(55 / 190)	0.29	52.6%(20 / 38)	49.5%(94 / 190)	0.82	21.0%(8 /38)	21.6%(41 /190)	0.15
Middle 33%	27.9%(17/ 61)	23.1%(49 / 212)	0.39	59.0%(36 / 61)	56.1%(119 / 212)	0.58	13.1%(8 / 61)	20.8%(44 / 212)	0.75
High 33%	25.0%(13 / 52)	24.4%(49 / 201)	0.24	55.8%(29 / 52)	57.7%(116 / 201)	0.34	19.2%(10 / 52)	17.9%(36 / 201)	0.94

#Measured on Likert scale

*Logistic regression, adjusted for baseline, age, gender and cluster

## Discussion

For (older) persons with complex care needs living in a community-dwelling setting where care is provided by multiple often autonomous professionals, person-centered and integrated care are widely accepted concepts. Particularly in geriatric care and primary care for older persons this is the case. Previous research has shown that this type of care is related to increased satisfaction in GP’s concerning organizational aspects of care and increased satisfaction in older persons concerning relational aspects of care.

In this study population of older persons with a high level of complexity of health problems, the overall satisfaction level did not differ after implementing person-centered integrated care as compared to usual care. Also, no relation was found between the levels of perceived health state and changes in satisfaction after implementation.

Not finding a marked effect on clinical outcomes and patient satisfaction after an intervention aimed at integrating and improving person-centered care for older persons is consistent with other studies. This can, amongst others, be attributed to an incomplete degree of implementation and/or a low contrast between the care in the intervention and control groups [12, 19, 31, 32]. As the degree of implementation of the intervention was not measured we cannot say with certainty whether this was the case in our study. However, since our study focuses on the relation between satisfaction and perceived health state and the non-significant variations in satisfaction in intervention and control groups showed no different pattern for the strata of perceived health state, we can conclude that this perceived health state does not act as an independent modifier of satisfaction.

A relationship has been reported before between other patient and care provider characteristics, and patient satisfaction. [14, 33] Particularly the interpersonal aspects of the care provider-patient interaction were found to be significant in relation to patient satisfaction. [34–36] However, we found no studies that further investigated the relation between the complexity of health problems, the perceived health state, and the satisfaction with care in older patients.

An earlier cross-sectional study found that the chance of *dis*satisfaction with the provided care increased with rising complexity of health problems. [13] However, the question remains whether this decreasing level of satisfaction was related mainly to the complexity of the health problems itself, or was also influenced by the perception of health state. In the aim to unravel this association, the present study focused on the perceived health state in older persons with a high level of complex problems. As expected, based on our earlier studies and literature, we found no significant effect on satisfaction after a change in the organization of care. Our finding that the various levels of perceived health state introduced no clear difference in change of satisfaction in the intervention vs. control group suggests that this is not an important modifier of patient satisfaction.

When regarding patient satisfaction as an indication of the fit of provided care, it should be taken into account that the level of complexity of health problems of the population influences patient satisfaction, and not the perceived health state. Therefore, the earlier found decreasing satisfaction with increasing complexity of health problems is more likely to be an indication of a patient-need versus care-organization discrepancy than of a negative state of mind of the patient.

Besides using patient satisfaction, person-centered, integrated care can also be evaluated by using the degree to which the personal goals of patients are achieved and the degree to which care organization is achievable and sustainable for the involved professional and informal care providers. The achievement of patient goals could be investigated, for example, by the method of ‘goal attainment scaling’ while the organizational aspects can be investigated by ‘mixed methods’ approaches and participatory ‘action research’ methods using frameworks such as the ‘realist approach’ and the ‘dynamic sustainability framework’ [37–39].

### Strengths

A strength of this study is that it provides a quantitative impression of the development of satisfaction in real-life implementation of person-centered integrated primary care. The trial design accommodates the reality of implementing improved care next to usual care.

The study also offers extensive in-depth data on the health state of the specific group of older persons with complex care needs; by using the TOPICS-CEP all these data have been combined and used. The TOPICS-CEP was developed and validated as an instrument to evaluate the quality of care for older persons and has since been validated for the concept ‘perceived health state’ in various populations. Being an aggregation of a number of clinical, functional and psychological study-instrument outcomes it has an area of overlap with concepts such as ‘care need complexity’, ‘multi-morbidity’ and ‘frailty’ [40]. However, being weighted by patient and informal caregiver preferences, with general wellbeing as a reference, it distinguishes itself from these other concepts. From a clinical viewpoint, we think that it provides a useful measure.

### Limitations

This study was performed in a population of older persons with self-reported problems in 3 and 4 out of 4 health domains. Therefore, it is a selected population of older persons with a high level of complexity of health problems with a decreased variation in experienced health state compared to the total population. Therefore, caution is required when generalizing these data to a population of older persons with a greater variation in level of complexity of health problems, as a greater variation in experienced health state may influence satisfaction to a differing extent.

As the intervention group was younger and better educated than the control group at baseline some inclusion bias could have occurred. This possible bias was corrected for by not comparing the actual satisfaction levels, but the changes within the intervention and control group.

The Likert scale is widely used in the evaluation of patient satisfaction. However, quantifying change using Likert data can be done in various ways. Due to the predominance of the middle options around ‘neutral’ and ‘satisfied’, the mean or median scores show little variation. As ‘very satisfied’ can be considered a meaningful expression of patient satisfaction, we used the percentage of respondents choosing this option as a measure of satisfaction level. To compensate for this limitation, we used an increase and decrease of at least 1 point on the Likert scale as an alternative.

### Conclusion and implications

We conclude that in these older persons with a high level of complexity of health problems, the satisfaction of GP care does not change during implementation of improved person-centered integrated care. In this relationship, the perceived health state does not act as an additional modifier. Therefore, the absence of a change in satisfaction must be seen more in relation to the expected and experienced care by the older persons than to their perceived state of health.

## References

[1] Waddington C, Egger D. Integrated Health Services—What and why? WHO [Internet]. 2008. Available from: http://www.who.int/healthsystems/technical_brief_final.pdf.

[2] Callahan CM. Controversies Regarding Comprehensive Chronic Care: Coordinated Care: The Drug-Free Wonder Drug. J Am Geriatr Soc. 2015;63(9):1938–40. 10.1111/jgs.13599 26338355

[3] Christensen K, Doblhammer G, Rau R, Vaupel JW. Ageing populations: the challenges ahead. Lancet. 2009;374(9696):1196–208. S0140-6736(09)61460-4 [pii]; 10.1016/S0140-6736(09)61460-4 19801098 PMC2810516

[4] James Lloyd SW. Integrated care. A guide for policy makers 2016 [updated 2016 14 june 2016; cited 2015 3/17/2015]. Available from: http://www.ilcuk.org.uk/images/uploads/publication-pdfs/pdf_pdf_7.pdf.

[5] Wise J. Services for older people need major change, says report. BMJ. 2014;348:g1994 10.1136/bmj.g1994 24609303

[6] C H, N W. Making integrated care happen at scale and pace: lessons from experience. London: The King's Fund 2013 [updated 2013; cited 2015 3/17/2015]. Available from: http://www.kingsfund.org.uk/publications/making-integrated-care-happen-scale-and-pace.

[7] Berwick DM, Nolan TW, Whittington J. The triple aim: care, health, and cost. Health affairs (Project Hope). 2008;27(3):759–69. Epub 2008/05/14. 10.1377/hlthaff.27.3.759 . 18474969

[8] UK Doh. Liberating the NHS:No decision about me, without me 2014 [updated 2014]. Available from: https://www.gov.uk/government/uploads/system/uploads/attachment_data/file/216980/Liberating-the-NHS-No-decision-about-me-without-me-Government-response.pdf.

[9] Williams B. Patient satisfaction: a valid concept? Soc Sci Med. 1994;38(4):509–16. 8184314 10.1016/0277-9536(94)90247-x

[10] Williams B, Coyle J, Healy D. The meaning of patient satisfaction: an explanation of high reported levels. Soc Sci Med. 1998;47(9):1351–9. S0277953698002135 [pii]. 9783878 10.1016/s0277-9536(98)00213-5

[11] Sitzia J, Wood N. Patient satisfaction: a review of issues and concepts. Soc Sci Med. 1997;45(12):1829–43. S0277953697001287 [pii]. 9447632 10.1016/s0277-9536(97)00128-7

[12] Kupfer JM, Bond EU. Patient satisfaction and patient-centered care: necessary but not equal. JAMA. 2012;308(2):139–40. 1216487 [pii]; 10.1001/jama.2012.7381 22782413

[13] Poot AJ, den Elzen WP, Blom JW, Gussekloo J. Level of satisfaction of older persons with their general practitioner and practice: role of complexity of health problems. PLoS One. 2014;9(4):e94326 10.1371/journal.pone.0094326 PONE-D-13-49650 [pii]. 24710557 PMC3978057

[14] Salisbury C, Wallace M, Montgomery AA. Patients' experience and satisfaction in primary care: secondary analysis using multilevel modelling. BMJ. 2010;341:c5004 10.1136/bmj.c5004 20940212 PMC2954274

[15] Jaques H. Putting patients at the heart of quality. BMJ. 2012;344:e3164 10.1136/bmj.e3164 22577188

[16] Berwick DM. What 'patient-centered' should mean: confessions of an extremist. Health Aff (Millwood). 2009;28(4):w555–w65. hlthaff.28.4.w555 [pii]; 10.1377/hlthaff.28.4.w555 19454528

[17] Reckrey JM, Soriano TA, Hernandez CR, DeCherrie LV, Chavez S, Zhang M, et al The team approach to home-based primary care: restructuring care to meet individual, program, and system needs. J Am Geriatr Soc. 2015;63(2):358–64. 10.1111/jgs.13196 25645568 PMC4780315

[18] Schrijvers G. Integrated Care—Better and Cheaper. Sutton, Surrey, UK: Reed Business Information; 2016. 292 p.

[19] Blom J, den Elzen WP, van Houwelingen AH, Heijmans M, Stijnen T, Van den Hout W, et al Effectiveness and cost-effectiveness of a proactive, goal-oriented, integrated care model in general practice for older people. A cluster randomised controlled trial: Integrated Systematic Care for older People-the ISCOPE study. Age Ageing. 2016;45(1):30–41. afv174 [pii]; 10.1093/ageing/afv174 26764392 PMC4711660

[20] Drewes YM, Blom JW, Assendelft WJ, Stijnen T, den Elzen WP, Gussekloo J. Variability in vulnerability assessment of older people by individual general practitioners: a cross-sectional study. PloS one. 2014;9(11):e108666 Epub 2014/11/08. 10.1371/journal.pone.0108666 25379778 PMC4224322

[21] Schellings R, Kessels AG, ter Riet G, Sturmans F, Widdershoven GA, Knottnerus JA. Indications and requirements for the use of prerandomization. Journal of clinical epidemiology. 2009;62(4):393–9. Epub 2008/12/06. 10.1016/j.jclinepi.2008.07.010 . 19056237

[22] Schellings R, Kessels AG, Ter Riet G, Kleijnen J, Leffers P, Knottnerus JA, et al Members of research ethics committees accepted a modification of the randomized consent design. Journal of clinical epidemiology. 2005;58(6):589–94. Epub 2005/05/10. 10.1016/j.jclinepi.2004.11.021 . 15878472

[23] Pascoe GC. Patient satisfaction in primary health care: a literature review and analysis. Eval Program Plann. 1983;6(3–4):185–210. 10299618 10.1016/0149-7189(83)90002-2

[24] Collins K, O'Cathain A. The continuum of patient satisfaction—from satisfied to very satisfied. Soc Sci Med. 2003;57(12):2465–70. S0277953603000984 [pii]. 14572851 10.1016/s0277-9536(03)00098-4

[25] Hofman CS, Makai P, Boter H, Buurman BM, de Craen AJ, Olde Rikkert MG, et al Establishing a composite endpoint for measuring the effectiveness of geriatric interventions based on older persons' and informal caregivers' preference weights: a vignette study. BMC Geriatr. 2014;14:51 1471-2318-14-51 [pii]; 10.1186/1471-2318-14-51 24742136 PMC4021341

[26] The National Care for the Elderly Programme. The National Care for the Elderly Programme [Internet]. 2014 3/17/2015. Available from: http://www.nationaalprogrammaouderenzorg.nl/english/the-national-care-for-the-elderly-programme/.

[27] Hofman CS, Makai P, Blom JW, Boter H, Buurman BM, Olde Rikkert MG, et al Comparing the health state preferences of older persons, informal caregivers and healthcare professionals: a vignette study. PloS one. 2015;10(3):e0119197 Epub 2015/03/05. 10.1371/journal.pone.0119197 25739034 PMC4349801

[28] Hofman CS, Lutomski JE, Boter H, Buurman BM, de Craen AJ, Donders R, et al Examining the construct and known-group validity of a composite endpoint for The Older Persons and Informal Caregivers Survey Minimum Data Set (TOPICS-MDS); A large-scale data sharing initiative. PloS one. 2017;12(3):e0173081 Epub 2017/03/16. 10.1371/journal.pone.0173081 28296910 PMC5351849

[29] Kempen GI, Miedema I, Ormel J, Molenaar W. The assessment of disability with the Groningen Activity Restriction Scale. Conceptual framework and psychometric properties. Social science & medicine (1982). 1996;43(11):1601–10. Epub 1996/12/01. . 8961404 10.1016/s0277-9536(96)00057-3

[30] Folstein MF, Folstein SE, McHugh PR. "Mini-mental state". A practical method for grading the cognitive state of patients for the clinician. Journal of psychiatric research. 1975;12(3):189–98. Epub 1975/11/01. . 1202204 10.1016/0022-3956(75)90026-6

[31] Chang JT, Hays RD, Shekelle PG, MacLean CH, Solomon DH, Reuben DB, et al Patients' global ratings of their health care are not associated with the technical quality of their care. Ann Intern Med. 2006;144(9):665–72. 144/9/665 [pii]. 16670136 10.7326/0003-4819-144-9-200605020-00010

[32] Metzelthin SF, van RE, de Witte LP, Ambergen AW, Hobma SO, Sipers W, et al Effectiveness of interdisciplinary primary care approach to reduce disability in community dwelling frail older people: cluster randomised controlled trial. BMJ. 2013;347:f5264 10.1136/bmj.f5264 24022033 PMC3769159

[33] Heje HN, Vedsted P, Sokolowski I, Olesen F. Patient characteristics associated with differences in patients' evaluation of their general practitioner. BMC Health Serv Res. 2008;8:178 1472-6963-8-178 [pii]; 10.1186/1472-6963-8-178 18715502 PMC2533311

[34] Berkelmans PG, Berendsen AJ, Verhaak PF, van der Meer K. Characteristics of general practice care: what do senior citizens value? A qualitative study. BMC Geriatr. 2010;10:80 1471-2318-10-80 [pii]; 10.1186/1471-2318-10-80 21044316 PMC2984451

[35] Bowling A, Rowe G, McKee M. Patients' experiences of their healthcare in relation to their expectations and satisfaction: a population survey. J R Soc Med. 2013;106(4):143–9. 106/4/143 [pii]; 10.1258/jrsm.2012.120147 23564898 PMC3618164

[36] Butterworth JE, Campbell JL. Older patients and their GPs: shared decision making in enhancing trust. Br J Gen Pract. 2014;64(628):e709–e18. 64/628/e709 [pii]; 10.3399/bjgp14X682297 25348995 PMC4220232

[37] Goodman C, Dening T, Gordon AL, Davies SL, Meyer J, Martin FC, et al Effective health care for older people living and dying in care homes: a realist review. BMC Health Serv Res. 2016;16:269 10.1186/s12913-016-1493-4 [pii]. 27422733 PMC4947336

[38] Timpel P, Lang C, Wens J, Contel JC, Gilis-Januszewska A, Kemple K, et al Individualising Chronic Care Management by Analysing Patients' Needs—A Mixed Method Approach. International journal of integrated care. 2017;17(6):2 Epub 2018/03/29. 10.5334/ijic.3067 29588635 PMC5854149

[39] Chambers DA, Glasgow RE, Stange KC. The dynamic sustainability framework: addressing the paradox of sustainment amid ongoing change. Implementation science: IS. 2013;8:117 Epub 2013/10/04. 10.1186/1748-5908-8-117 24088228 PMC3852739

[40] Fried LP, Tangen CM, Walston J, Newman AB, Hirsch C, Gottdiener J, et al Frailty in older adults: evidence for a phenotype. The journals of gerontology Series A, Biological sciences and medical sciences. 2001;56(3):M146–56. Epub 2001/03/17. . 11253156 10.1093/gerona/56.3.m146

